# Selecting implementation strategies to improve implementation of integrated PrEP for pregnant and postpartum populations in Kenya: a sequential explanatory mixed methods analysis

**DOI:** 10.1186/s43058-023-00481-9

**Published:** 2023-08-14

**Authors:** Sarah Hicks, Ben Odhiambo, Felix Abuna, Julia C. Dettinger, Nancy Ngumbau, Laurén Gómez, Joseph Sila, George Oketch, Enock Sifuna, Bryan J. Weiner, Grace John-Stewart, John Kinuthia, Anjuli D. Wagner

**Affiliations:** 1 Department of Epidemiology, University of Washington, Seattle, WA USA; 2 Kenyatta National Hospital, Nairobi, Kenya; 3 Department of Global Health, University of Washington, Seattle, WA USA; 4 Department of Health Systems and Population Health, University of Washington, Seattle, WA USA; 5 Departments of Medicine, University of Washington, Seattle, WA USA; 6 Departments of Pediatrics, University of Washington, Seattle, WA USA

**Keywords:** HIV infections/prevention and control, Humans, Pregnancy and postpartum, Strategy prioritization, Nominal group technique

## Abstract

**Background:**

There is a higher risk for HIV acquisition during pregnancy and postpartum. Pre-exposure prophylaxis (PrEP) is recommended during this period for those at high risk of infection; integrated delivery in maternal and child health (MCH) clinics is feasible and acceptable but requires implementation optimization.

**Methods:**

The PrEP in Pregnancy, Accelerating Reach and Efficiency study (PrEPARE; NCT04712994) engaged stakeholders to prioritize determinants of PrEP delivery (using Likert scores) and prioritize PrEP delivery implementation strategies. Using a sequential explanatory mixed methods design, we conducted quantitative surveys with healthcare workers at 55 facilities in Western Kenya and a stakeholder workshop (including nurses, pharmacists, counselors, and county and national policymakers), yielding visual plots of stakeholders’ perceived feasibility and effectiveness of the strategies. A stepwise elimination process was used to identify seven strategies for empirical testing. Facilitator debriefing reports from the workshop were used to qualitatively assess the decision-making process.

**Results:**

Among 146 healthcare workers, the strongest reported barriers to PrEP delivery were insufficient providers and inadequate training, insufficient space, and high volume of patients. Sixteen strategies were assessed, 14 of which were included in the final analysis. Among rankings from 182 healthcare workers and 44 PrEP policymakers and implementers, seven strategies were eliminated based on low post-workshop ranking scores (bottom 50th percentile) or being perceived as low feasibility or low effectiveness for at least 50% of the workshop groups. The top seven strategies included delivering PrEP within MCH clinics instead of pharmacies, fast-tracking PrEP clients to reduce waiting time, delivering PrEP-related health talks in waiting bays, task shifting PrEP counseling, task shifting PrEP risk assessments, training different providers to deliver PrEP, and retraining providers on PrEP delivery. All top seven ranked strategies were grouped into bundles for subsequent testing. Facilitator debriefing reports generally aligned with rankings but noted how stakeholders’ decision-making changed when considering the impact of strategies on facility staff and non-PrEP clients.

**Conclusions:**

The most impactful barriers to integrated PrEP delivery in MCH clinics were insufficient staffing and space. Implementation strategies prioritized through multiple methods of stakeholder input focused on co-location of services and increasing clinic efficiency. Future testing of these stakeholder-prioritized strategy bundles will be conducted to assess the effectiveness and implementation outcomes.

**Supplementary Information:**

The online version contains supplementary material available at 10.1186/s43058-023-00481-9.

Contributions to the literature

- Pre-exposure prophylaxis (PrEP) is a recommended intervention for pregnant women at risk for HIV, but implementation is sub-optimal
- Stakeholders involved in the delivery and receipt of PrEP can provide valuable insights on implementation barriers and strategies that clinics can test to better meet the needs of PrEP clients.
- The most impactful barriers were insufficient staffing and space. Using three strategy prioritization methods, stakeholders prioritized co-location, task shifting, and flow modifications as implementation strategies to address these barriers.
- These findings set the foundation for testing the impact of stakeholder-prioritized PrEP delivery strategies on implementation outcomes.

## Introduction

Women are at elevated risk for HIV acquisition during pregnancy and the postpartum period [1, 2]. Women diagnosed with HIV during pregnancy and the postpartum period disproportionately contribute to infant HIV infections globally [3–5]. For these at-risk individuals, pre-exposure prophylaxis (PrEP) provides a woman-controlled and risk-period-specific method of HIV prevention in the form of a once-daily oral pill [6, 7]. PrEP is recommended by WHO and Kenyan guidelines during pregnancy and postpartum [8, 9]. Several previous and ongoing studies have demonstrated that PrEP use in pregnancy is safe [10–13]. However, despite potential benefits and a track record of safety, PrEP is often not provided for perinatal women, even in high-burden settings, highlighting gaps in implementation.

Several projects have been implemented in Kenya to provide PrEP for pregnant and postpartum women [14–16]. Qualitative work among adolescent girls and young women in Kenya demonstrated that motherhood was a central reason for initiating PrEP [17]. Among these women, PrEP was considered helpful to protect their children and remain healthy to fulfill their responsibilities to their families [17]. These findings highlighted the need for integrated maternal and child health (MCH) and PrEP services. Two projects delivered integrated PrEP within MCH clinics across Kenya’s public health sector [15, 16, 18–22]*.* In one of these studies, qualitative focus group discussions were conducted among 50 healthcare workers (HCWs) offering PrEP in MCH and family planning (FP) clinics [21]. Using the Consolidated Framework for Implementation Research (CFIR) as a guide, participants were asked about the perceived benefits and challenges of implementing PrEP in MCH clinics as well as potential strategies to overcome the implementation barriers [21]. HCWs felt that PrEP delivery in MCH clinics would be highly feasible and acceptable as it would improve PrEP coverage and decrease stigma compared to PrEP delivered in HIV care clinics [21]. However, HCWs also noted several implementation challenges, such as increased workload for MCH staff due to documentation and increased demand for HIV testing services, physical space constraints, drug stockouts, and competing priorities with implementing partners [21]. HCWs identified a range of implementation strategies that could possibly overcome these barriers, such as task shifting HIV testing, fast tracking PrEP clients, conducting PrEP health talks to increase demand, and enhancing provider education on PrEP [21]. While some MCH clinics have organically implemented various combinations of these strategies, there has been no formal prioritization or testing of the strategies’ ability to overcome the implementation barriers identified by HCWs.

Using the RE-AIM evaluative framework, a systematic review identified planned, ongoing, or existing studies that address determinants and test strategies for delivering integrated PrEP into MCH clinics, categorizing determinants in the social-ecological model [23]. Included studies focused primarily on strategies that address individual- and provider-level determinants [23]. Of the included studies in this review that had results, standardized patient actor trainings improved counseling quality, and universal offer of PrEP was found to be superior to risk-guided PrEP offer [24, 25]. At the individual level, PrEP uptake was found to be higher following point-of-care sexually transmitted infection testing [26]. However, few studies have tested strategies that address facility- and program-level determinants which are akin to system-level determinants within the socioecological model, such as facility space constraints and suboptimal human resources to address PrEP demand in MCH [23]. In the present analysis, we quantitatively investigated prioritization of these determinants and strategies and used strategy prioritization methods to identify three PrEP delivery strategy bundles to pilot and evaluate in Kenyan MCH clinics. This analysis will provide future program implementers with an overview of the prioritization process with a diverse group of PrEP stakeholders, as well as insights into the most promising strategies for PrEP integration in MCH at a variety of socioecological levels.

## Methods

### Study design

The *PrEP in Pregnancy, Accelerating Reach and Efficiency* (PrEPARE; NCT04712994) study gathered qualitative and quantitative data from stakeholders to identify determinants of PrEP implementation and PrEP implementation strategies. This analysis of data from the PrEPARE study is a sequential explanatory mixed methods evaluation of three strategy prioritization methods and one method for strategy grouping [27]. A mixed methods approach was used to holistically understand the strategies that stakeholders preferred for integrated PrEP delivery in MCH and their reasons for selecting those strategies. Data was collected sequentially over time. Past experience surveys and the strategy bundling exercise were completed from October 2020 to July 2021; the post-workshop rankings and go-zone plots were collected during a workshop in August 2021.

### Study setting and participants

This study was conducted in three counties in Kenya which rank among the top 5 Kenyan counties for high HIV prevalence (9% or higher): Kisumu, Homa Bay, and Siaya Counties [28]. For the past experience surveys, data was collected from HCWs with prior experience delivering PrEP to pregnant and postpartum populations at one of the 55 study facilities. The post-workshop rankings and go-zone plot data were collected in Kisumu County at an in-person stakeholder workshop, including PrEP policymakers, implementers, and other national- and county-level officials from the Kenyan Ministry of Health (Additional file 1). Other key stakeholders (PrEP users, HCWs, and representatives from the County and National AIDS and STD Control Program (NASCOP)) were purposively sampled and identified through existing networks. All participants were ≥ 18 years.

### Data collection

#### Implementation determinants and implementation strategy past experience survey

Previous qualitative work using CFIR helped identify barriers to PrEP implementation and potential strategies to address these barriers [21, 29]. To assess the determinants’ perceived impact and HCW’s previous experience using mitigating strategies, a survey was administered in-person, over telephone, or online through REDCap with PrEP-experienced HCWs. First, participants ranked the barriers based on their personal experiences working in facilities on a 1–5 Likert scale to assess their impact (“none” to “strong”) on PrEP delivery. Participants were also asked to rate 16 PrEP delivery strategies based on what had been tried at their facility and the perceived strength of influence that strategy had on improving PrEP delivery. Strategies were rated with the following values: “tested and improved delivery,” “tested but did not improve delivery,” and “did not test.”

#### Implementation strategy prioritization workshop surveys and go-zone plots

The stakeholder workshop utilized the nominal group technique (NGT), a group prioritization method that democratizes decision-making [30, 31]. The NGT begins with group generation of ideas, individual ranking of the generated strategies, and small group discussions to gather group consensus. Workshop participants were placed in cadre groups to minimize potential power imbalances and focus stakeholder expertise on strategies relevant to their experience. In small groups, participants were asked to rank each strategy on a 5-point Likert scale for perceived feasibility and effectiveness. Go-zone plots were created in R Studio based on individuals’ ratings to facilitate group discussions [32, 33]. Each group’s mean feasibility (*x*-axis) and effectiveness (*y*-axis) scores were plotted; strategies whose mean feasibility and effectiveness scores each averaged 2.5 or higher fell within the “go-zone” in the top-right quadrant of the go-zone. Afterwards, participants were asked to respond to an online REDCap survey and individually rank a set of 16 PrEP delivery strategies on their perceived effectiveness. In these post-workshop rankings, strategies were sequentially ranked from 1 (most effective) to 16 (least effective).

#### Implementation strategy prioritization workshop small group qualitative notes

The Kenyan study staff with experience delivering PrEP in MCH clinics were trained as small-group facilitators for the workshop activities. Facilitators compiled notes during the small group discussions to track the pros and cons of each strategy’s feasibility and effectiveness and took notes on the small groups’ overall reflections about each strategy after viewing the go-zone plots, including additional group suggestions for implementation considerations. We utilized debriefing reports, completed by discussion facilitators, to evaluate the small group decision-making process. The debriefing reports followed a structured format to collect key takeaway messages from the small group discussions; in a previous evaluation of structured debriefing reports, they were found to accurately reflect the key thematic content from full in-depth interview transcripts [34]. During data analysis and construction of this manuscript, small group facilitators were consulted to provide additional insights into participants’ discussion and ensure participants’ views were accurately reported.

### Data analysis

Descriptive statistics were calculated by summarizing continuous variables using medians and interquartile ranges. Categorical variables were summarized using sample sizes (*n*) and percentages. To determine the top three PrEP delivery strategy bundles, all 16 strategies were first ranked using the past experience surveys from PrEP-experienced HCWs. Rankings were determined by the percentage of HCWs who indicated that the strategy had been tested at their facility and improved PrEP delivery. Strategies were then excluded if they ranked in the bottom 50% of post-workshop strategy rankings or fell outside the go-zone for the majority of stakeholders. The 50% cutoff point was subjectively selected to determine the strategies that fell above and below the median ranking and to limit the number of strategies for testing. In the post-workshop rankings, strategies’ scores were averaged across participants. Strategies were also excluded if they fell outside of the go-zone for more than 50% of the go-zone plots in which it was evaluated to identify the strategies that fell above the median ranking. All data were analyzed using R version 4.2.2.

Following the quantitative data collection during the stakeholder workshop, we utilized the facilitator debrief reports to gain insight into participants’ rationale for each strategy’s rating, using the qualitative data in a building function with the quantitative [35]. To analyze the facilitator debriefing reports, we used the Framework Method, a targeted content analysis approach that uses deductive categories based on a conceptual model [36]. The Framework Method was chosen over an in-depth coding approach as the qualitative data was utilized to supplement findings from the quantitative analysis, and rich, nuanced themes were not the focus of this analysis. Additionally, the highly deductive coding approach used and the rapidity of analysis are well suited to a matrix format of the Framework Method [36]. The categories used in this analysis were pros and cons of feasibility and effectiveness, overall group reflections on each strategy, and additional considerations for each strategy’s implementation. A matrix was created in Excel to organize and synthesize small group discussions into these categories. A second reviewer evaluated the matrix, and a consensus approach was used to resolve any discrepancies in feasibility and effectiveness categorization.

### Reporting guidelines

A completed copy of the Strengthening the Reporting of Observational studies in Epidemiology (STROBE) guidelines for cross-sectional studies can be found in Additional file 2 [37].

## Results

A total of 185 HCWs were invited to complete the past experience survey, of which 183 completed the survey. There were 48 PrEP stakeholders invited to participate in the workshop, of which 46 attended; 44 participants (91.7%) completed the pre-small-group and go-zone plot rankings while 40 (83.3%) completed the post-small-group rankings. Participant demographics are described in Table 1. Demographic characteristics were similar among both PrEP-experienced HCWs and the stakeholder workshop participants. In both populations, slightly more than half of the participants identified as female (62.8% and 56.5%, respectively). The median age of participants was 32 among HCWs and 40 among workshop participants. Nearly all participants reported attending a college or university (95.6% and 93.5%, respectively), and participants had spent a median of 2.3 and 3 years providing PrEP to individuals of any age among HCWs and workshop participants, respectively. For the past experience surveys, 185 HCWs were invited to participate, and 182 (98.4%) respondents completed the surveys; the 55 facilities that they worked at represented a range of settings, with a majority from rural areas, public institutions, and Kisumu county.

**Table 1:** Participant demographics

Characteristic	PrEP-experienced healthcare workers (*N* = 183), *n* (%) or median (IQR)	Workshop participants (*N* = 46), *n* (%) or median (IQR)
Female	115 (62.8)	26 (56.5)
Age in years	32 (29, 38)	40 (34, 46)
Highest educational attainment		
None	0 (0)	0 (0)
Primary	0 (0)	0 (0)
Secondary	2 (1.1)	2 (4.4)
Polytechnic	6 (3.3)	1 (2.2)
University/college	175 (95.6)	43 (93.5)
Current employment role		
Clinical officer	23 (12.6)	6 (13.0)
Counselor (peer, general, or social worker)	6 (3.3)	1 (2.2)
In-charge	31 (16.9)	4 (8.7)
Nurse or nurse counselor	87 (47.5)	6 (13.0)
Research staff	22 (12.0)	NA
Doctor	0 (0)	3 (6.5)
Pharmacist	NA	3 (6.5)
Partner representative	NA	4 (8.7)
AIDS and STI control program (county, sub-county, or national)	NA	7 (15.2)
Others	14 (7.7)	12 (26.1)
Years providing PrEP to individuals of any age	2.3 (1.5, 3.3)	3 (1.0, 4.0)
Years providing care to pregnant or postpartum women (*N* = 182)	4.3 (3.1, 7.1)	–
Years providing PrEP to pregnant or postpartum women	2.3 (1.5, 3.3)	–
Received training to pregnant or postpartum women	125 (68.3)	–
Received training in providing PrEP adherence counseling to pregnant or postpartum women	113 (61.8)	–

**Fig. 1:**
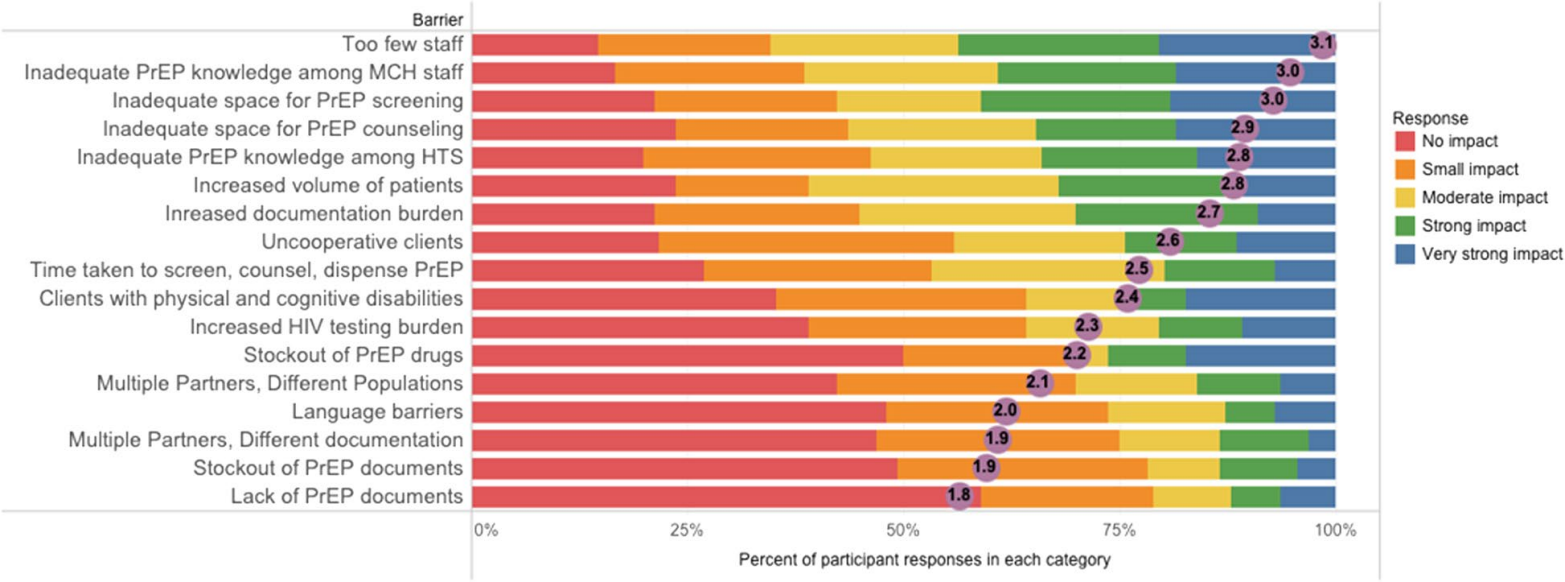
PrEP implementation determinants from the past experience survey

In the past experience surveys, HCWs identified determinants that had a high impact on the ability to deliver PrEP: insufficient provider-patient ratios, inadequate provider training, lack of physical space for PrEP services, and documentation burden. Barriers that had little to no impact on PrEP delivery included PrEP and document stockouts, HIV testing burden, competing partner priorities, and language barriers (Fig. 1).

To prioritize strategies for implementation and testing as part of the PrEPARE study, 16 strategies identified through prior qualitative work were evaluated using the three different prioritization approaches [21]. However, two strategies were dropped from the analysis. “Coordination with adolescent-friendly services” was erroneously grouped with “provision of communication aids” in the go-zone analysis, and small group facilitators were instructed to direct participants to only discuss communication aids. “Task shifting any other component of PrEP counseling, assessment, or dispensing” was also dropped as it was erroneously excluded from the go-zone plot discussions. The final rankings presented below only include the 14 strategies that had official ranks between all three data sources.

**Fig. 2:**
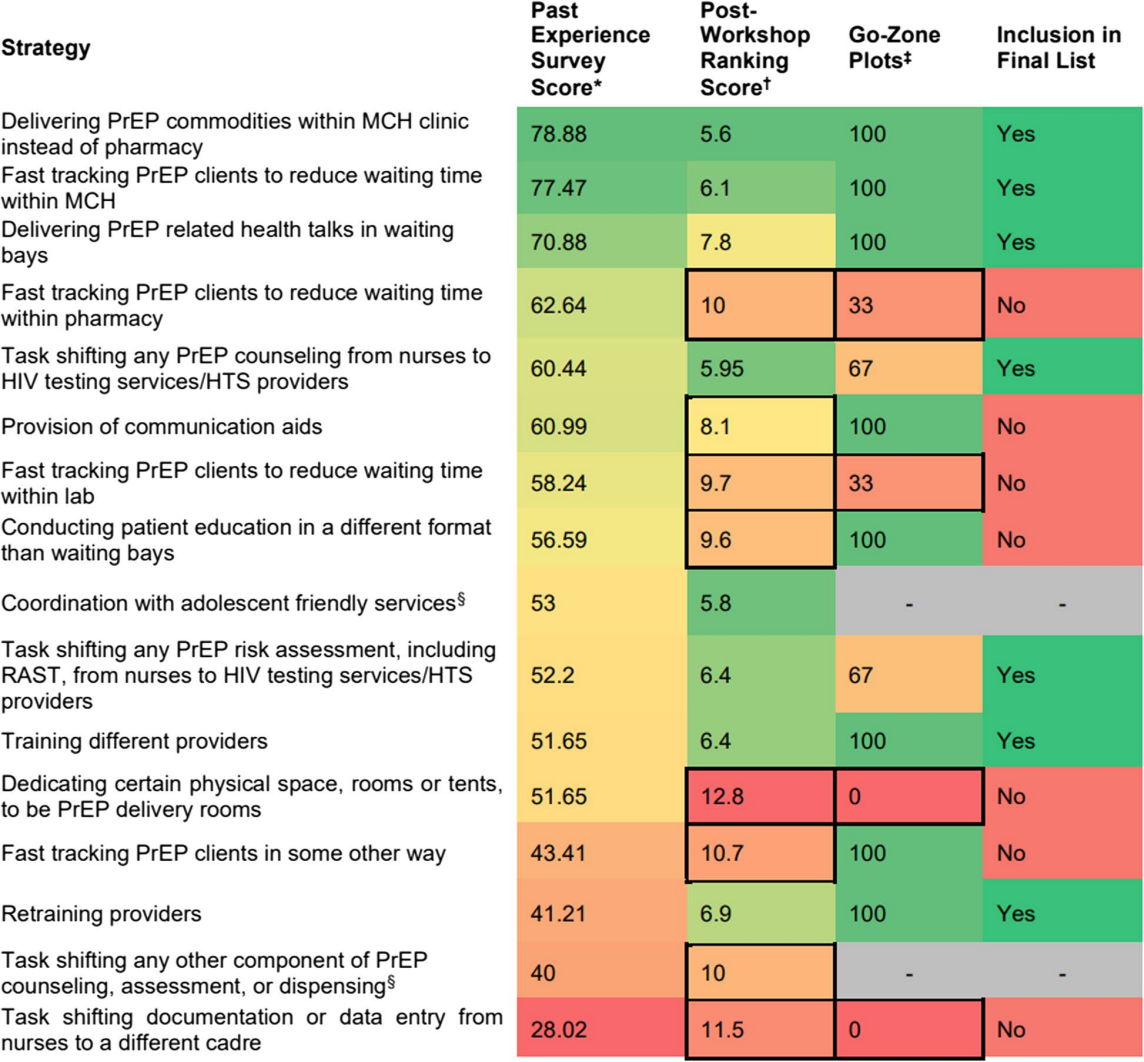
Identification of highly ranked strategies from three strategy ranking methods. *Percentage of PrEP-experienced healthcare workers reporting the strategy was tested at their facility and improved PrEP delivery. ^†^Mean post-workshop ranking on a scale of 1–16. ^‡^Proportion of groups that rated the strategy in the go-zone for both feasibility and effectiveness scores (%). ^§^This strategy was not evaluated during the go-zone plot creation

Seven PrEP delivery strategies were selected utilizing the three prioritization methods (Fig. 2). Seven strategies were eliminated from the list based on low post-workshop ranking scores (bottom 50th percentile) or falling outside the go-zone plot for at least 50% of the workshop groups. Of these seven eliminated strategies, four were excluded using the criteria from both the post-workshop rankings and go-zone plot analyses while three were excluded based on low post-workshop rankings alone. The go-zone plots did not exclude any additional strategies, and the two strategies whose scores were missing for the go-zone plots were also previously excluded by the post-workshop rankings.

**Fig. 3:**
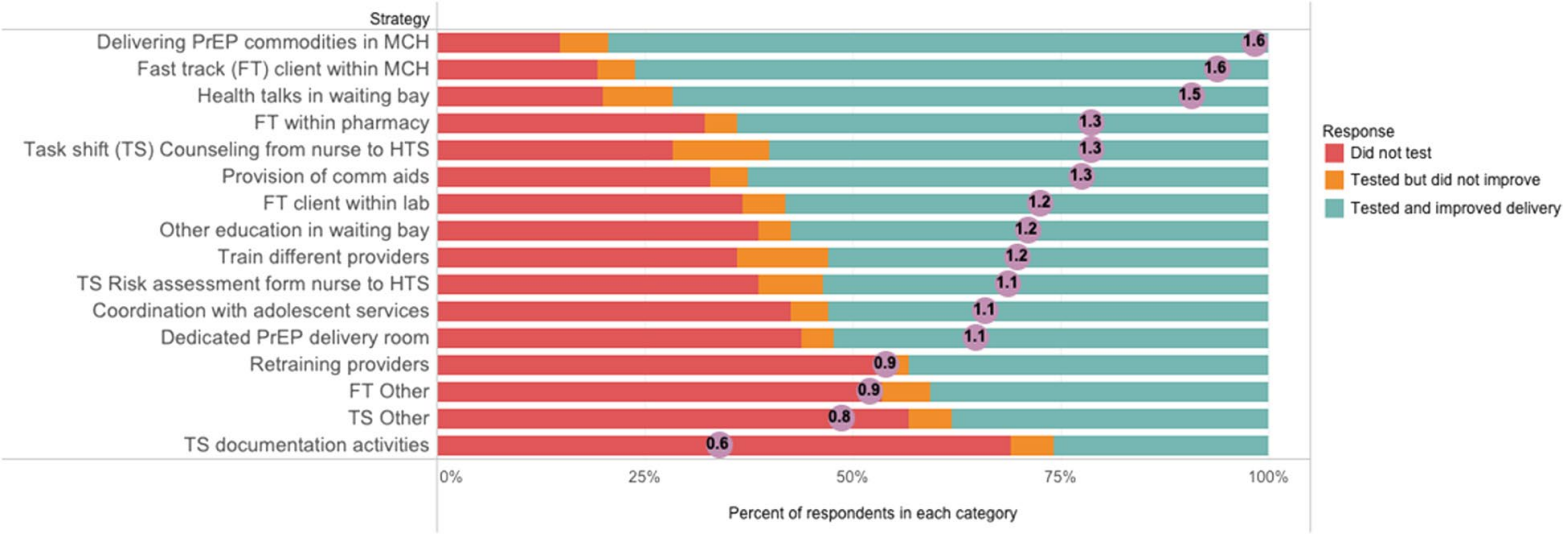
Distribution of previously tested strategies from the past experience survey

The highest scoring strategies using the past experience surveys were “delivering PrEP commodities within MCH clinics instead of pharmacies,” “fast tracking PrEP clients to reduce waiting time within MCH,” and “delivering PrEP-related health talks in waiting bays.” These three strategies were also in the top 50th percentile for the go-zone plots and the post-workshop surveys. The remaining strategies had heterogeneous rankings across the three ranking approaches, without clear patterns. The “retraining providers” strategy was the second lowest ranked strategy in the past experience surveys but highly ranked in both the go-zone and post-workshop survey and therefore included in the final list of strategies to be tested; importantly, 48% of PrEP-experienced healthcare workers indicated that this strategy had not been tested at their facilities (Fig. 3).

The summary of facilitator notes is provided (Additional file 3). Overall, small group discussions of the pros and cons of each strategy’s feasibility and effectiveness aligned with the strategies’ rankings in the go-zone plots. For example, group members who discussed “delivering PrEP commodities within MCH clinics instead of pharmacy” described the benefits of this strategy as reducing waiting times for clients, reduced drop-out rates and improved PrEP uptake, increased client satisfaction with services, and reduced stigma; these findings mirror the high feasibility and effectiveness ratings that this strategy received. The participants did note some drawbacks to this strategy such as increased MCH nurse workload and the potential difficulties of documenting drug dispensing, but the detailed list of effectiveness pros for this strategy left the groups feeling that this strategy was very feasible and effective. In notable contrast, strategies that were rated with very low feasibility and effectiveness scores such as “task shifting documentation” and “dedicating certain physical spaces as PrEP delivery rooms” had significantly longer lists of feasibility and effectiveness cons in the debriefing reports.

The group facilitator notes also offered insight into the thought processes behind the small groups’ ranking decisions in the go-zone plots. For example, “fast tracking clients in MCH clinics” was ranked number 1 in the pre-workshop rankings but fell to number 9 in the go-zone plot rankings. The facilitator debriefing reports indicated that this strategy would place an undue burden on MCH clinic staff and that this model of service delivery would unfairly disadvantage clients who are not receiving PrEP by increasing their wait times. Conversely, the facilitator notes on “retraining providers,” which was ranked number 10 in the pre-workshop rankings and number 3 in the go-zone plots, highlighted several strong pros for feasibility and effectiveness with relatively few cons. The participants felt that this would be easy to implement using computer-based training programs and that a skills-based training would improve overall service delivery quality at the implementing sites. The small group discussions about each of the strategies offered significant room for strategy ranking changes through consideration of how these strategies would impact the facility staff and all client groups.

### Strategies to be tested in MCH based on the strategy priorities identified

The identified top strategies will be tested in a series of interrupted time series studies in MCH clinics in the next 2 years (K01MH121124). A total of seven strategies were selected and grouped for three rounds of future testing within MCH clinics. The first set of strategies includes “fast tracking PrEP clients” and “retraining providers on PrEP”; the second and third sets respectively include “task shifting PrEP counseling to HIV testing services (HTS) providers” and “training different providers” as well as “delivering PrEP-related health talks in waiting bays” and “providing communication aids.” Delivering PrEP in MCH clinics will be tested in all three rounds as this strategy was the most highly ranked. Audit and feedback of data systems will also be provided per the request of clinic staff. Each round of testing involves an additional two strategies which were grouped together after discussion with the study staff. Furthermore, a recent large cluster randomized trial by our team revealed that risk scores provide no additional benefit in appropriate allocation of PrEP, suggesting that risk score-guided PrEP should not be prioritized for continuation in Kenya [25]. As a result, “task shifting any PrEP risk assessment, including Rapid Assessment Screening Tool (RAST), from nurses to HIV testing services/HTS providers” was removed from the list of potential strategies to continue with.

## Discussion

The three strategy prioritization methods used in this study enabled the identification of seven unique strategies that may be feasible and effective for delivering PrEP into MCH clinics. Overall, PrEP-experienced HCWs identified several substantial barriers to PrEP implementation that fall under the Implementation Process Domain of CFIR including insufficient provider-patient ratios, lack of provider training, and documentation burden [38]; the highest ranked strategies across all prioritization methods will be able to alleviate their impact. For example, inadequate provider training can be addressed through retraining providers on PrEP and training different providers to assist in PrEP delivery. Task shifting and fast tracking could ameliorate the burden on HCWs to integrate PrEP in MCH clinics. The Expert Recommendations for Implementing Change (ERIC) strategies are a set of implementation strategies compiled via a systematic review to improve the consistency of strategy reporting; the strategies prioritized in this study aligned with the ERIC strategies of revising professional roles (task shifting), developing and distributing educational materials, conducting educational meetings (communication aids and health talks), and changing service sites (delivering PrEP and health talks in MCH) [39]. The two populations surveyed in this study demonstrated good agreement on overall strategy prioritization.

In a recent systematic review on PrEP in pregnancy and implementation science, the Reach, Efficacy, Adoption, Implementation, and Maintenance (RE-AIM) Framework was used to evaluate the current scientific evidence in PrEP implementation through determinants, outcomes, and strategies at the individual, provider, facility, and program levels [23, 40]. Of the 12 completed, ongoing, and planned studies in this review, eight focused on individual determinants, including demographic and behavioral risk factors and perceived risk for HIV acquisition [15, 22, 23]. Three ongoing studies looked at individual-level determinants of implementation while a fourth planned study looked at implementation determinants at the facility level [23]. In our prior qualitative study, HCWs noted many barriers to PrEP implementation in MCH clinics, including workload and documentation for staff, physical space constraints, drug stockouts, competing partner priorities, and increases in HIV testing [21]. These provider- and facility-level barriers generally aligned with the perceived barriers identified in the surveys in our present study. However, HCWs in this survey indicated that PrEP stockouts, competing priorities, and HIV testing were less impactful barriers to successful PrEP implementation than other barriers, findings that provide a useful prioritization-oriented complement to the qualitative information. There is a need for studies to test provider- and facility-level strategies to address these barriers. Most studies planned to test strategies that would improve individual PrEP uptake through targeted counseling and other behavioral interventions [23]. One study sought to test a new provider training model using role-playing with patient-actors, but no other studies tested or planned to test strategies to address PrEP implementation determinants in health systems [23]. The PrEPARE study provides a unique perspective by combining the experience of diverse stakeholders to prioritize strategies that will address provider- and facility-level determinants such as task shifting, fast-tracking, and improving provider education on PrEP. While the strategies identified in this study have not yet been tested for impact on integrated PrEP delivery, several have been demonstrated to be effective in other settings and contexts [41–46]. For example, a study in the Democratic Republic of the Congo showed that ART fast tracking services improved patient retention [41]. Similarly, task shifting has been shown to be an effective strategy in HIV care; one systematic review found that task shifting ART counseling and delivery results in improved clinic efficiency, maintained or improved quality of care, reduced client waiting times, and reduced loss to follow-up [42]. A cluster RCT of ART task shifting in South Africa showed no differences in mortality or virological outcomes [43]. Provider training has had mixed efficacy in previous studies; when provided with task shifting, training different providers on ART delivery demonstrated increased uptake with no change in the quality of care [42]. Similarly, training in adolescent-friendly care resulted in increased retention in one study [44]. However, a systematic review from sub-Saharan Africa indicated limited improvement in antenatal care with training [45]. This conflicting evidence highlights the need for specialized training on HIV-related topics and further studies to assess the impact of retraining teams on peripartum PrEP delivery. Additionally, few studies have evaluated the efficacy of providing health talks specific to PrEP and HIV in clinic waiting bays despite the fact that this is recommended [47]. Qualitative work with HCWs who have participated in a recent clinical trial involving PrEP for HIV prevention showed that this strategy was preferred by PrEP-experienced HCWs [46].

The facilitator notes from the debriefing reports aligned with the strategies’ feasibility and effectiveness score ratings as well as the overall go-zone rankings. However, it is important to note that there are different cultural interpretations of the words “feasibility” and “effectiveness.” In this study, feasibility was described as, “How easy would it be to do strategy X?”, and effectiveness was described as, “How much of an impact would strategy X have?”. These descriptions were chosen instead of using the Feasibility of Intervention Measure after consultation with teams who had experience adapting descriptions of implementation outcomes in a Kenyan setting [48, 49]. Implementation science measures and terminology have been primarily developed in the USA and Canada, and the understanding of these words and concepts must be considered when applying implementation science terminology in other settings. For example, one study that evaluated 33 agencies in 9 countries found 29 unique terms that were used to refer to the implementation science concept of “knowledge to action” [50]. The heterogeneity with which these terms are used has the potential to lead to great confusion and a lack of generalizable findings in the field as a whole [51]. Efforts to adapt and validate psychometric evaluations in different cultural contexts offer a promising example of cross-cultural adaptation of implementation science terminology [52–54].

Our study has several strengths. First, we evaluated the perspectives of a diverse group of stakeholders. Collecting strategy rankings from PrEP-experienced healthcare workers in addition to from policymakers and individuals living with HIV enabled us to incorporate diverse perspectives regarding what will be feasible and effective to implement. The past experience rankings survey is, to date, the largest data collection effort in the world that focuses on healthcare workers’ experiences in PrEP in pregnancy. Obtaining feedback from a variety of stakeholders allowed us to take a more holistic approach in evaluating which strategies should be implemented and evaluated as part of the PrEPARE study. The use of the democratic process in achieving stakeholder consensus is a unique contribution of this study.

Our study has several limitations. First, the past experience rankings from healthcare workers may have had recall bias. There were power differences between the stakeholder workshop participants who were national- and county-level officials, healthcare workers, and PrEP users. These power differences may have stifled discussion and potentially introduced social desirability bias, but the NGT was used to democratize decision-making by giving all group members an equal voice in go-zone plot creation. Additionally, cadre groups were assigned a set of strategies that best aligned with their experiences and expertise to ensure that all participants could make meaningful contributions to the small group discussions. Each of the three strategy ranking methods used during the stakeholder workshop was also conducted individually with participants to minimize social desirability bias. The main limitation of this analysis is that we did not formally match strategies and barriers, nor did we seek to prioritize strategies based on previously identified barriers. Some natural groupings of strategies and barriers emerged post hoc, but this was not considered in the analysis a priori. While it is not possible to know how our results would have been different if we had utilized barrier-strategy matching methods—stemming from a lack of empiric comparison data in the literature—we theorize that we might have selected strategies that more closely aligned with commonly experienced barriers or those that had a high critical impact on service delivery. During the workshop, participants were invited to suggest any additional strategies during the pre-workshop surveys; suggestions primarily included increased adherence counseling and community sensitization and delivery of PrEP. We did not incorporate these two suggestions into the go-zone plot surveys or discussions due to (1) the technical challenge of incorporating new strategies real-time into the go-zone plots production and (2) the suggested strategies were either a specific topic (adherence counseling) covered under other included strategies or a demand generation rather than facility-based service provision strategy (community sensitization). This approach differed from the first step of a traditional NGT, limiting direct methodologic comparison. Purposive sampling was used to recruit participants for the stakeholder workshop. While purposive sampling is not statistically representative, the goal of this study was to gain knowledge from content experts and understand stakeholder perspectives on PrEP implementation in MCH clinics. Finally, our team has a companion manuscript that includes a comparison of methodologic performance and pragmatism between the prioritization methods utilized in this study [55].

## Conclusions

This study sought to identify three bundles of PrEP delivery strategies to implement and test in Kenyan MCH clinics. Through engagement with PrEP-experienced healthcare workers, we identified several high-priority barriers to integrating PrEP delivery into MCH clinics including insufficient staff and space, inadequate provider knowledge of PrEP, and increased volumes of patients. Through the past experience surveys and the workshop activities, we identified a set of seven strategies that might address these barriers. Strategies included task shifting and fast tracking to increase clinic efficiency and reduce provider workload as well as retraining current providers and training new providers to bolster knowledge of PrEP and quality of care across services. The use of health talks in waiting bays and communication aids also seek to improve the efficiency of patient-provider communication regarding PrEP. Future testing of these strategies will allow for the assessment of effectiveness and implementation outcomes at the individual, provider, and facility levels.

### Supplementary Information

**Additional file 1.** Summary of study designs, settings, subjects, and data collection across data sources.

**Additional file 2.** STROBE Statement—Checklist of items that should be included in reports of cross-sectional studies.

**Additional file 3.** Facilitator notes from the PrEPARE stakeholder workshop.

## Data Availability

The datasets generated and/or analyzed during the current study are not publicly available due to the limitations in data sharing permissions from the Kenyan ethical review boards but are available from the corresponding author upon reasonable request. The datasets used and analyzed during the current study are available from the corresponding author upon reasonable request.

## Abbreviations

CFIR: Consolidated Framework for Implementation Research
ERC: Ethics and Research Committee
HCW: Healthcare worker
HIV: Human immunodeficiency virus
HTS: HIV testing services
IRB: Institutional Review Board
KNH-UoN: Kenyatta National Hospital-University of Nairobi
MCH: Maternal and child health
NASCOP: National AIDS and STD Control Program
NGT: Nominal group technique
PrEP: Pre-exposure prophylaxis
PrEPARE: PrEP in Pregnancy, Accelerating Reach and Efficiency
RAST: Rapid Assessment Screening Tool
RE-AIM: Reach, Efficacy, Adoption, Implementation, and Maintenance Framework
UW: University of Washington
WHO: World Health Organization
