# Supplementary material for: Selecting implementation strategies to improve implementation of integrated PrEP for pregnant and postpartum populations in Kenya: a sequential explanatory mixed methods analysis
Source: Implement Sci Commun. 2023 Aug 14;4:93. doi: 10.1186/s43058-023-00481-9 (PMC10424426; doi:10.1186/s43058-023-00481-9)
Supplement: Supplementary file 1 — Additional file 1. Summary of study designs, settings, subjects, and data collection across data sources. [file 43058_2023_481_MOESM1_ESM.docx]

Additional file 1: Summary of study designs, settings, subjects, and data collection across data sources

|  | **Surveys with PrEP-Experienced HCWs** | | **Stakeholder Workshop** | | |
| --- | --- | --- | --- | --- | --- |
|  | **Past Experience Rankings** | **Strategy Bundling Exercise** | **Pre-Small-Group Rankings** | **Go-Zone Plots** | **Post-Small-Group Rankings** |
| Study design | Quantitative cross-sectional design | | Quantitative cross-sectional design | Qualitative and quantitative cross-sectional design | Quantitative cross-sectional design |
| Study setting | 55 facilities in Kisumu, Homa Bay, and Siaya Counties | | One, in-person workshop at Grand Royal Swiss Hotel, Kisumu | | |
| Study subjects | Healthcare workers (HCWs) working at a facility involved in 2 prior PrEP delivery projects/studies; ≥18 years | | PrEP policymakers, PrEP implementers, frontline healthcare workers involved in PrEP delivery and decision-making; ≥18 years | | |
| Data collection | Online surveys | | Online surveys | Facilitated discussions; online surveys | Online surveys |
| Ranking/rating approach | 3-point, categorical Likert scale (Tested but did not improve; Did not test; Tested and improved);  N = 183 | Concept mapping groupings;  N = 183 | Sequential strategy rankings from 1 (least effective) to 16 (most effective);  N = 44 | 5-point Likert scale for strategies’ perceived feasibility and effectiveness;  N = 44 | Sequential strategy rankings from 1 (least effective) to 16 (most effective);  N = 40 |
