# Supplementary material for: Selecting implementation strategies to improve implementation of integrated PrEP for pregnant and postpartum populations in Kenya: a sequential explanatory mixed methods analysis
Source: Implement Sci Commun. 2023 Aug 14;4:93. doi: 10.1186/s43058-023-00481-9 (PMC10424426; doi:10.1186/s43058-023-00481-9)
Supplement: Supplementary file 3 — Additional file 3. Facilitator notes from the PrEPARE stakeholder workshop. [file 43058_2023_481_MOESM3_ESM.docx]

Additional file 3: Facilitator notes from the PrEPARE stakeholder workshop

| Strategy  (Rank 1-14) | FEASIBILITY | | EFFECTIVENESS | | Overall Group Reflections | Additional Thoughts on Implementation |
| --- | --- | --- | --- | --- | --- | --- |
|  | PROS | CONS | PROS | CONS |  |  |
| Waiting bays  (1) | -Addresses many clients at same time  -Easy to include PrEP health education in normal routine health talks that are done at MCH  -Once trained, everyone in MCH can provide health information to clients | -Needs to be done at the right time to reach most people  -Needs structure, otherwise people will pick the information they like  -Requires training for mentor mothers and CHVs  -Cannot be in every waiting bay  -Late comers will miss health talks if it’s only offered during morning hours | -Creates PrEP demand  -Increases sensitization  -Increase knowledge, uptake, and adherence  -Could use brochures/voucher system to invite male partners to the clinics  -Decrease stigma | -May spread misinformation if talk is not structured | - All the members agreed that this is feasible in most of our facility set ups | -Focus on times with high client flow  -Can use the services of CHVs and CHWs who understand PrEP  -Have structured format or flip charts already filled with main points to standardize delivery of health talk  -Ensure capacity building of staff so they have PrEP knowledge  -Include HIV self-testing kits at the waiting bays as uptake of HIVST and PrEP go together  -Start from the basics and use simple language  -Use HTS providers/clinical team to give talks (not peer educators) |
| Dispensing PrEP in MCH  (2) | -Reduce waiting time  -One stop shop system | -Difficult documentation of dispensed drugs  -Increase MCH nurse workload | -Reduces drop-outs through improved follow-up  -Enhances discussions between service provider and clients receiving other services  -Improve PrEP uptake  -Increase client satisfaction  -Reduce stigma  -Minimize back-and-forth movement of clients | - Increase MCH nurse workload  -Varied waiting time between clients who are and are not seeking PrEP  -May compromise quality | - The group agreed that it was very feasible and effective | -Need to pilot this strategy in some periphery and mid-level facilities  -Integrate or merge MCH clinic appointment date with PrEP visit  -Task share (e.g. with mentor mother) |
| Re-train providers  (3) | -Can use electronic models  -Efficiency of service delivery based on most recent knowledge | -Lack of trained personnel  -No time for training | -Could be skills based  -Lead to quality service delivery |  | - Easy to implement with a huge impact | -The trainings should be skill based and use more of experience sharing and electronic models including Benchmarking.  -Avoid classroom-like training  -Include CHVs in risk assessment and champions of demand creation  -Target the us of in-service training, CMEs, and webinars with award certificates to encourage attendance |
| Communication aids  (4) | -Materials available on NASCOP website (cost effective)  -Standardized coming from national materials  -Available in local languages  -More feasible in high resource settings  -Saves time by equipping clients with necessary PrEP information  -Already on-going though with minimal intensity | -Resources for IEC dissemination are limited  -Difficult to maintain as adolescents are transitory | -Create knowledge/ awareness  -Dispel myths and misconceptions  -Can bring youth together but not for PrEP service delivery | -Massive resource requirement for personnel to produce comm. Aids, offer services, and engage adolescents | - One of the best strategies that majority of the members feel that is easy to implement and may have enormous impact in the population especially the youth | -Create boards and wall hangings with various information and messages  -Use social media and other modern applications that are more accessible to youth (Google Apps)  -Engage the Ministry in developing policy papers for Adolescent Reproductive health service delivery with retaining of HCWs |
| Train different providers  (5) | -Can incorporate into on-the-job training  -Decrease client wait time  -Enhance staff capacity building  -Encourage ownership of services/ideas by all HCWs  -Can be implemented through on-job trainings/ mentorship  -Facility sensitizations and CMEs are existing avenues to utilize  -Easily engage MCH clinic peer educators, mentor mothers, and youth advocates | -Needs financial inputs  -Lack of trained personnel  -Low funds to facilitate training  -Sustainability is a challenge  -Limited privacy with lack of working space in some facilities  -Stigma may increase in case other clients realize the purpose of the visit | -Could make PrEP 24-hour service  -Facilitates demand creation  -Increase staff knowledge/service provision  -Increases coverage when most providers have required knowledge  -Improve commodity management  -Low cadre staff availability in facilities  -Effective training modalities  -Proper linkage to care for specialized needs | -Lack of trained personnel  -Lack of space  -Confidentiality breach may be more likely if using low cadre staff | - Majority of the members agreed that the strategy will be feasible to implement and will have positive impact | -PrEP should not be made a healthcare issue therefore all cadres should be trained including in advocacy by schoolteachers and churches.  -Train target groups for specific training areas  -Promote/facilitate on-the-job training  -Facilitate training by the county in collaboration with implementing partners to enhance sustainability  -Encourage exchange visits for bench marking to areas where this has worked for replication |
| Fast tracking other  (6) | -Reduces wait time for clients (including through integration of PrEP services)  -Integrates PrEP services to reduce lost clients | -Limited human resources (registration needs to escort clients to PrEP service delivery point)  -Requires additional training  -Not feasible with current resources  -May result in lack of documentation (missing indicators in registers) | -Works best for follow-up clients or those who know they are at risk and need PrEP  -Reduce stigma | -Non-PrEP clients will be discouraged  -Reduce sense of responsibility by healthcare workers |  | -Use PrEP cards to help identify clients easily  -Use community ART/PrEP refill groups  -Link PrEP clients to support groups  -Link PrEP clients to CHVs to help follow-up with PrEP clients in the community |
| Patient education in a format other than waiting bays  (7) | -Good for high volume hospitals  -Saves time by educating clients  -Can use CHVs during home visits  -Could be done as task shifting where each healthcare worker at every service delivery point offers health talks about PrEP to the clients | -Requires more space and time  -Requires human resources and training  -Involving CHVs would require more financial incentives | -Increase uptake/retention (especially for mothers who opted to take PrEP)  -Reduce stigma | -Client may not feel comfortable with 1:1 communication | - Members agreed that it was very feasible and effective  - Will improve PrEP Uptake and retention but not to a bigger /larger extent | -Identify potential PrEP clients and have group education session with them  -Introduce phone counseling and education programs  -Introduce one-on-one education with mothers at healthcare delivery points  -Health talks are more acceptable when given in waiting bays |
| Task shifting RAST from nurses to HTS providers  (8) | -HTS already doing this  -Alignment of PrEP/HIV assessment tools  -Enhance rapport and confidentiality assurance  -No replication of assessment | -Requires training  -HTS have other targets from partners  -Loss of PrEP-eligible clients between ANC/PNC and HTS rooms  -Inadequate HTS providers currently  -HTS providers cannot carry out physical/lab tests to determine PrEP-eligibility  -Conflict with partners and employers’ targets | -Save time for patients (PrEP delivery and HIV testing services)  -Improve client retention/ confidentiality  -Establishing rapport between client and HTS improves uptake  -Reduces workload  -Improve client retention because services will be provided by one person (easier tracking/follow-up) | -Role conflicts with clinician assessments  -HTS providers may feel they are not meeting stipulated partners/employers set targets | - Majority agreed that the strategy is very feasible and will be very effective in the MCH  - feasible however will have minimal impact and not very easy to implement because the HTS providers are employed by partners and have different priorities and targets therefore might lead to missed opportunities. | - Should be handled well to avoid role conflict.  -Encourage task sharing in facilities with inadequate staffing of HTS providers  -Train and facilitate HTS providers with necessary tools to effectively RAST clients  -Counseling and RAST should be done together |
| Fast tracking MCH  (9) | -Works well with DSD (differentiated service delivery) since they have a leader who picks the drugs on behalf of the clients and distributes them  -Helps MCH staff work as team  -Link PrEP clients more easily | -Differs from ‘first come, first served’ model clients know  -May reduce time keeping of PrEP clients since they know they will be served first  -May require extra labor force | -Improve uptake and retention  -Reduced wait time for clients | -Increase workload for those working at reception  -HCW conflict because waiting time is too long for other clients who are not on PrEP | - Members agreed the that it would be very feasible and effective |  |
| Task shift prep counseling from nurses to HTS providers (10) | -HTS already involved  -Solves staff shortage  -Reduce nurse workload  -Reduce client waiting time in MCH  -Less financial burden with current HTS involvement | - Extra work for HTS  - Partners control staff roles  -Nurse/clinician roles are not well defined  -Inadequate HTS providers in most facilities  -May interfere with MCH service delivery integration  -Sustainability when partners withdraw is a challenge | -Reduce client wait time  -Reduce nurse workload  -Increase uptake screening services/PrEP | -Requires training for HTS  -May miss some clients if nurses assume HTS will screen them | - Majority of the team agreed that the strategy is feasible and will bring positive impact in PrEP service provision in the MCH  -Has a high impact if implemented but not easy to implement. | - This strategy should not be called task shifting because the counsellors will see this as somebody else’s activities added to them. It should be christened ‘counsellor expanded mandate’ to avoid this.  -Focus on task sharing rather than shifting to ensure continuity of service delivery  -Task shift both risk assessment and counseling rather than separating the two  -Define the specific activities that the nurses/clinicians will be required to undertake with regards to PrEP services  -Train/capacity building for the HTS providers on PrEP services delivery/ counseling  -Recruitment of more HTS providers in the health facilities |
| Fast tracking lab  (11) | -Reduced turnaround time of investigations  -Reduced stigma as clients don’t know why a person visited the lab | -Several queuing sessions before delivery  -Requires more financial resources  -Reagents are costly  -Difficult to maintain ISO standards and commodities  -Basic tests we use are usually not done  -Few facilities currently offer lab services  -No guarantee of commodity security | -Requires additional personnel | -Lack of available personnel  -No space for additional lab staff  -Could increase stigma when labs are segregated and labeled  -Confidentiality may decrease if more people interact with a client  -Most clients don’t go through labs  -Increase waiting time for other clients  -Ensuring sample collection is within our control, but receipt of results is out of our control |  | -Engage partners fully to operationalize the service delivery  -HIV testing/urinalysis can be fast tracked, but other tests like creatine are more difficult |
| Task shifting documentation  (12) | -Reduce documentation workload | -May create errors; person who performs procedures should document  -Lack of personnel and partner priorities  -General documentation (monthly reports for routine services) is already challenging  -May lead to disintegrated paperwork and blanks in registers | -Effective data collection | -Teamwork needed is hard to achieve  -Reduce data quality more so in family planning and MCH clinics.  -Collected data not currently used in decision making | -Feasible however will have minimal impact and not very easy to implement because the HTS providers are employed by partners and have different priorities and targets therefore might lead to missed opportunities. | -The advent of electronic documentation will also pose a challenge to this strategy if implemented.  -Awareness creation on data collection use and planning to enhance partner participation in documentation  -Review of the registers to ensure no missed opportunities or double entries. |
| Fast tracking pharmacy  (13) | -Saves time (prepacks can be prepared in advance)  -May work better in mini pharmacies (eg MCH pharmacies)  -One stop shop at MCH  -Fast tracking of ARVs done in CCC, so we can use the same model | -All clients still need pharmacy services  -Requires extra manpower to escort clients  -Increase wait time for non-prep clients |  | -Increase stigma  -Confusion as stable patients are prioritized over sick patients  -Increased defaulters’ discontinuation and loss to follow-up | -The impact will be high but this strategy cannot be applied with ease.  - Members agreed that this strategy is feasible but had reservation on its effectiveness as other clients may know why these clients are served first. This may lead to stigma. | -Drugs could be prepacked  -Should apply this strategy in MCH pharmacies, not main pharmacies.  -Use model for fast tracking of ARVs done in CCC  -Efficient delivery requires task shifting of PrEP from pharmacy to MCH  -Sensitize pharmacists to identify PrEP clients  -Modify MOH protocols (ie first come first serve) |
| Dedicating physical spaces as PrEP delivery rooms  (14) | -Ensure privacy  -Decongest MCH clinics  -Capacity building opportunity with CHVs | -Increase stigma  -Not feasible due to staffing shortages  -Lead to missed opportunities with clients  -Expensive  -Facilities prefer other services to get new rooms | -Ensures privacy  -Could increase PrEP uptake/adherence in facilities with sufficient resources for a special room | -Not effective in achieving eMTCT targets  -Increase stigma  -Lower adherence and PrEP linkages | - Majority agreed that the strategy will not be feasible to implement and may have negative impact on the PrEP service provision in the MCH  - General view among all members were that this strategy is less feasible.  - Members agreed that having a special room won’t have a bigger positive impact due to the reason that patient will feel stigmatized to be seen going to the “PrEP room”. | - Integration of PrEP services within the MCH. This could be done by partitioning rooms to provide privacy  -Would not work unless using a post-natal room that is not labeled  -When creating a special room or tent for PrEP delivery, that facility should give it a unique name and not just label is as “PrEP Room” as a way of reducing stigma |
| General Recommendations across strategies:   - Need to empower CHV’s through trainings on PrEP in order for them to make referrals and educate at the community level - Need to include the community to improve PrEP initiation - Integration should be done rather than task shifting. - Have partners also prioritize PrEP delivery service package - Local government should consider employing HTS providers or absorbing those employed to ensure sustainability of services | | | | | | |
